# Reflecting on 2022

**DOI:** 10.1371/journal.pbio.3001957

**Published:** 2022-12-16

**Authors:** Joanna Clarke, on behalf of the PLOS Biology Staff Editors

**Affiliations:** 1 Public Library of Science, San Francisco, California, United States of America and Cambridge, United Kingdom

## Abstract

As 2022 draws to a close, we look back at some of the recent changes that have taken place at PLOS Biology, highlight some of our editors’ favorite moments from the past year across the life sciences, and thank our editors, authors and peer-reviewers.

2022 will be a memorable year for many reasons. The war in Ukraine, rising living costs across vast swathes of the world, and the ongoing COVID-19 pandemic with all its repercussions have cast a shadow over the past year, but there have been reasons for hope too. The UN Ocean Conference ended with several pledges and commitments for action that it will be important to see through to completion and, although not reaching the needed agreement about fossil fuel use, COP27 at least concluded with an agreement from wealthier nations to provide compensation to those nations most affected by climate-related disasters. Elsewhere in the life sciences, environmental DNA opened a window into the past, enabling us to reconstruct ancient ecosystems, AI enabled us to predict the structure of almost every known protein, and, following controversies in the field [1], trial results suggest a new anti-amyloid drug may finally be able to slow cognitive decline in Alzheimer’s disease.

At *PLOS Biology*, 2022 brought several changes too. Our editorial board grew to include 263 academic researchers across 29 countries as we said goodbye to 25 Academic Editors and welcomed 72 new ones. Our neuroscience editor Gabriel Gasque also sadly left us to take on an exciting new role at protocols.io. In his place, we welcomed Kris Dickson to the team, who has brought with her a wealth of knowledge about scientific publishing and all aspects of neuroscience. We were also fortunate to host Suzanne de Bruijn as an editorial intern for part of the year. You can learn more about her experience with us in a blog post she wrote for the *PLOS Biologue*, which has also played host this year to a new series of “Behind the paper” blog posts from authors of recently published studies, telling the stories of the twists and turns that took their research projects in new directions.

Another big change in 2022 came in our magazine section. I joined the journal at the beginning of 2022 as the magazine section editor and have enjoyed getting to grips with the amazing variety of topics we cover in our Perspectives, Community Pages, Essays, and Unsolved Mysteries. This year, we published our first Consensus Views, a new article type for the magazine section. These pieces provide guidelines or recommendations on topics of wide interest to the life sciences community, and have so far included topics as varied as recommendations for optimal levels of light exposure for human health [2] and ideas for ways to empower early career researchers to improve research culture [3].

As the magazine section editor, I also have the privilege of overseeing special collections of articles that aim to raise awareness of specific events or issues. In 2022, we published a collection on Mendel’s legacy in modern genetics to mark the 200^th^ birthday of Gregor Mendel, and a collection on Ocean solutions for a sustainable, healthy and inclusive future to explore potential solutions to mitigate the impacts of human activity on ocean ecosystems in line with the UN Ocean Decade. We already have plans in place for some exciting collections in 2023, so watch this space!

As well as launching a new type of magazine article, 2022 also saw the publication of our first Pre-Registered Research Articles (PRAs, also known as Registered Reports) [4]. For these articles, the peer review is split into two phases; one that happens before the experiments are conducted, and one that happens once the data has been analyzed and the paper has been written. It is hoped that this approach will improve the quality of research and reduce the focus on ‘flashy’ results and storytelling in publications. This year we published PRAs on decision-making [5] and spinal cord injury [6], and hope to see many more studies across the breadth of the life sciences published using this approach in the near future.

2022 also saw a return to in-person attendance at conferences. As editors, we have particularly relished being able to meet researchers again face-to-face and catch up with the exciting new developments in our respective fields after such a long break. That said, virtual conference attendance has also been a boon for us, enabling us to dip our toes into unfamiliar topics and learn from and interact with researchers from parts of the globe that are often underrepresented at scientific meetings. We attended around 40 meetings in 2022 and are busy planning our conference schedule for 2023, so do look out for us at meetings you might be attending.

Last but not least, 2022 saw the publication of some excellent science. We have chosen a selection of our favorite *PLOS Biology* papers from this year as some recommended reading to see you through into the New Year (Box 1).

**Box 1. PLOS Biology editors’ picks from 2022**

**Luke Smith (stem cell biology, neurodegeneration, physiology, epigenetics, and circadian rhythms)**

My choice is “Complementary encoding of spatial information in hippocampal astrocytes” by Curreli et al [7]. This study contributes to an emerging body of work indicating that astrocytic calcium signaling may regulate neuronal function and animal behaviors, with the authors reporting that astrocytic calcium signals encode spatial information in a virtual spatial navigation task. This line of research suggests that information encoding extends beyond neuronal circuits, and it will be exciting to see further work tease apart exactly how and if encoding in astrocytes modulates behavior and circuit activity.

**Roli Roberts (genetics, evolutionary biology, ecology, behavior, and meta-research)**

My choice is “Kelp carbon sink potential decreases with warming due to accelerating decomposition” by Filbee-Dexter et al [8]. The authors shipped standardized mesh bags of kelp to 12 different regions of the Northern hemisphere and measured the rate of decomposition and its relationship to environmental temperature, enabling them to predict the likely change in kelp’s carbon sink potential for the rest of this century. This study shows that while kelp decomposition will accelerate at low latitudes, there is scope for increased carbon sequestration by encouraging kelp forest growth at higher latitudes.

**Kris Dickson (neuroscience)**

My choice is “The microbiota promotes social behavior by modulating microglial remodeling of forebrain neurons” by Bruckner et al [9]. This study demonstrates that host-associated microbiota can affect brain development and alter social behavior in zebrafish, contributing to a burgeoning field of science assessing crosstalk between host microbiota and nervous system function. The authors found the loss of diverse zebrafish-associated bacterial strains modulates the abundance and function of forebrain microglia during a critical developmental time window, with these animals showing subsequent deficits in their normal social behavioral repertoire. These findings are a first step towards an understanding of the complex relationships between host-associated microbiota and development and function of the brain in both health and neurodevelopmental disease states.

**Richard Hodge (molecular biology, structural biology, and biotechnology)**

My choice is “Mitochondria transplantation between living cells” by Gäbelein et al [10]. In this study, the authors present a method called FluidFM that can efficiently transplant mitochondria directly between live cells. The transplantation can be performed without prior treatment or depletion of mitochondrial DNA in the recipient cells. The technique could be used for a wide variety of experimental applications in the future, such as tracking mitochondrial variants, analyzing mitochondrial dynamics or even rejuvenating cells with low metabolic activity in stem cell therapies.

**Paula Jauregui (microbiology and immunology)**

My choice is “Widespread expression of the ancient HERV-K (HML-2) provirus group in normal human tissues” by Burn et al [11]. In this study, the authors found that a human endogenous retrovirus that has previously been associated with disease is also expressed in healthy tissue. This study highlights the importance of virus-derived proteins as normal components of the human proteome in many tissues and suggests that they may contribute to physiological functions yet to be discovered.

**Ines Alvarez-Garcia (cell biology, signaling, development, aging, and plant biology)**

My choice is “Erebosis, a new cell death mechanism during homeostatic turnover of gut enterocytes” by Ciesielski et al [12]. This study proposes a new cell death mechanism for Drosophila gut enterocytes that the authors term ‘erebosis’. They provide evidence suggesting that this is a distinct form of cell death that is conserved in mammals, including humans, and propose that erebosis maintains intestinal tissue homeostasis during physiological cell turnover. The preliminary nature of the data makes it perfect for our Discovery Report format, and future studies will hopefully identify the erebotic genes that regulate this exciting new process.

All that remains is to thank the authors, reviewers and Academic Editors who have contributed to *PLOS Biology* in 2022 and without whom this journal would not exist, to thank you for reading, and to hope that 2023 brings more excellent science that can change the future for the better, for all of us.
